# Beta-blockers have no impact on survival in pancreatic ductal adenocarcinoma prior to cancer diagnosis

**DOI:** 10.1038/s41598-020-79999-0

**Published:** 2021-01-13

**Authors:** Anthony Yang, Haley M. Zylberberg, Sheila D. Rustgi, Sunil P. Amin, Ariel Bar-Mashiah, Paolo Boffetta, Aimee L. Lucas

**Affiliations:** 1 Henry D Janowitz Division of Gastroenterology, Icahn School of Medicine at Mount Sinai, One Gustave Levy Place, Box 1069, New York, NY 10029 USA; 2 Division of Gastroenterology, University of Miami Leonard Miller School of Medicine, Miami, FL USA; 3 Tisch Cancer Institute, Icahn School of Medicine at Mount Sinai, New York, NY 10029 USA

**Keywords:** Cancer, Gastrointestinal cancer

## Abstract

Previous studies have suggested that β-adrenergic signaling may regulate the growth of various cancers. The aim of our study is to investigate the association between the incidental use of beta-blockers for various conditions on the overall survival of patients with pancreatic ductal adenocarcinoma (PDAC). Patients with histologically-confirmed PDAC between 2007 and 2011 were extracted from Surveillance, Epidemiology, and End Results registry (SEER)-Medicare linked database. Kaplan Meier and multivariable Cox Proportional-Hazard models were used to examine the association between beta-blocker usage before diagnosis and overall survival adjusting for appropriate confounders. As an additional analysis we also examined continuous beta-blocker use before and after diagnosis. From 2007 to 2011, 13,731 patients were diagnosed with PDAC. Of these, 7130 patients had Medicare Part D coverage in the 6-month period before diagnosis, with 2564 (36%) of these patients using beta-blockers in this period. Patients receiving beta-blockers had a mean survival time of 5.1 months compared to 6 months for non-users (p < 0.01). In multivariable analysis, beta-blockers usage was not associated with improved survival (Hazard Ratio (HR) 1.04, 95%, Confidence Interval (CI) 0.98–1.1, p = 0.2). When patients were stratified by conditions with indications for beta-blocker usage, such as hypertension, coronary artery disease and cardiac arrhythmia, differences in survival were insignificant compared to non-users in all groups (p > 0.05). After stratification by receptor selectivity, this lack of association with survival persisted (p > 0.05 for all). As a subgroup analysis, looking at patients with continuous Medicare Part D coverage who used beta-blockers in the 6-month period before and after cancer diagnosis, we identified 7085 patients, of which 1750 (24.7%) had continuous beta blocker use. In multivariable analysis, continuous beta-blockers usage was associated with improved survival (Hazard Ratio (HR) 0.86, 95%, Confidence Interval (CI) 0.8–0.9, p < 0.01). Beta-blocker usage before diagnosis does not confer a survival advantage in patients with PDAC, though continuous use before and after diagnosis did confer a survival advantage. Prospective studies into the mechanism for this advantage are needed.

## Introduction

Pancreatic cancer is the fourth most common cause of cancer-related death in the United States^1^. The 5-year survival rate for pancreatic cancer at all stages is a low eight percent with most pancreatic cancers being diagnosed at late stages with distant metastases, which is associated with a 5-year survival rate of 3%^1^. Additionally, the mortality rates for pancreatic cancers have been reported to rise annually by 0.3% in males from 2011 to 2015 with no change in females, contrasting with the considerable decline in mortality for the four most common cancers (breast, prostate, lung and colorectal) in the same time period^1^. Given the poor prognosis, research has focused on evaluating whether common medications may have a therapeutic benefit in the treatment of pancreatic cancer.

Preclinical studies have demonstrated the role of the beta-adrenergic signaling system in the pathogenesis of various cancers, including pancreatic cancer^2–6^. It has been shown that beta-adrenergic agonists cause the activation of protein kinase A and mitogen activated protein kinase (MAPK) pathways. Downstream effects of this pathway lead to activation of transcription factors that promote cell proliferation, including nuclear factor κB (NFκB) and cyclic-AMP (cAMP) response binding protein (CREB)^2,7^. Therefore, factors associated with higher catecholamine levels, such as beta-adrenergic agonists, chronic stress and smoking, have been shown to stimulate the growth and progression of cancers of various organs, including ovarian, breast, colon and pancreas^8^.

Thus, it would be expected that beta-adrenergic antagonists may have a potential role in inhibiting the progression of cancer. Indeed, previous preclinical studies have demonstrated a potential benefit in utilizing beta-blockers in various cancers, including ovarian, lung, colorectal and pancreatic cancer^7,9–12^. Beta-blockers can antagonize the β1 or β2 receptor and it has been demonstrated that antagonism of either receptor can potentially inhibit the invasion of pancreatic ductal adenocarcinoma (PDAC)^7^. An animal model study found that propranolol was effective in the prevention of ethanol-induced PDAC by blocking cAMP-dependent release of EGF and VEGF^10^.

Despite preclinical studies suggesting beta-blockers as a therapeutic strategy for cancer, there has been conflicting clinical evidence on the potential use of beta-blockers on pancreatic cancer^13–23^. Additionally, there have been reported differences in efficacy on survival between selective and non-selective beta-blockers for cancer^14^. It has been suggested that non-selective beta-blockers may have a greater effect on inhibiting cancer progression due to their ability to inhibit both the cAMP/PKA and Ras pathway compared to selective beta-blockers, which only inhibit the cAMP/PKA pathway^7,14^.

Beta-blockers are currently indicated for several common diseases such as hypertension, arrhythmias and heart failure, making their usage prevalent. Using a large national cancer database, we sought to determine the effect of the incidental use of beta-blockers for various conditions on the survival of patients with pancreatic ductal adenocarcinoma (PDAC). Additionally, differences in efficacy on cancer-specific survival between non-selective and selective beta-blockers will also be evaluated. Finally, the effectiveness of beta-blockers on survival will be evaluated across patients undergoing different pancreatic cancer therapies.

## Methods

### Data source

Data was obtained from the Surveillance, Epidemiology and End Results (SEER) registry linked to Medicare claims. The SEER program is a collection of cancer registry in various states, covering approximately 28% of the US population^24^. The registry contains patient-level information such as demographic characteristics, tumor characteristics, diagnostic confirmation, surgery, chemotherapy, radiation therapy and survival. Medicare is the primary health insurer for Americans aged 65 years and older. The Medicare claims data contains patient information, including demographics and claims for inpatient (Part A) and outpatient services (Part B). Data from Medicare Part D, which contains information on prescription drug coverage beginning in 2007, was also available, which limited this study to patients diagnosed in 2007.

### Patient population and medication usage

**Figure 1:**
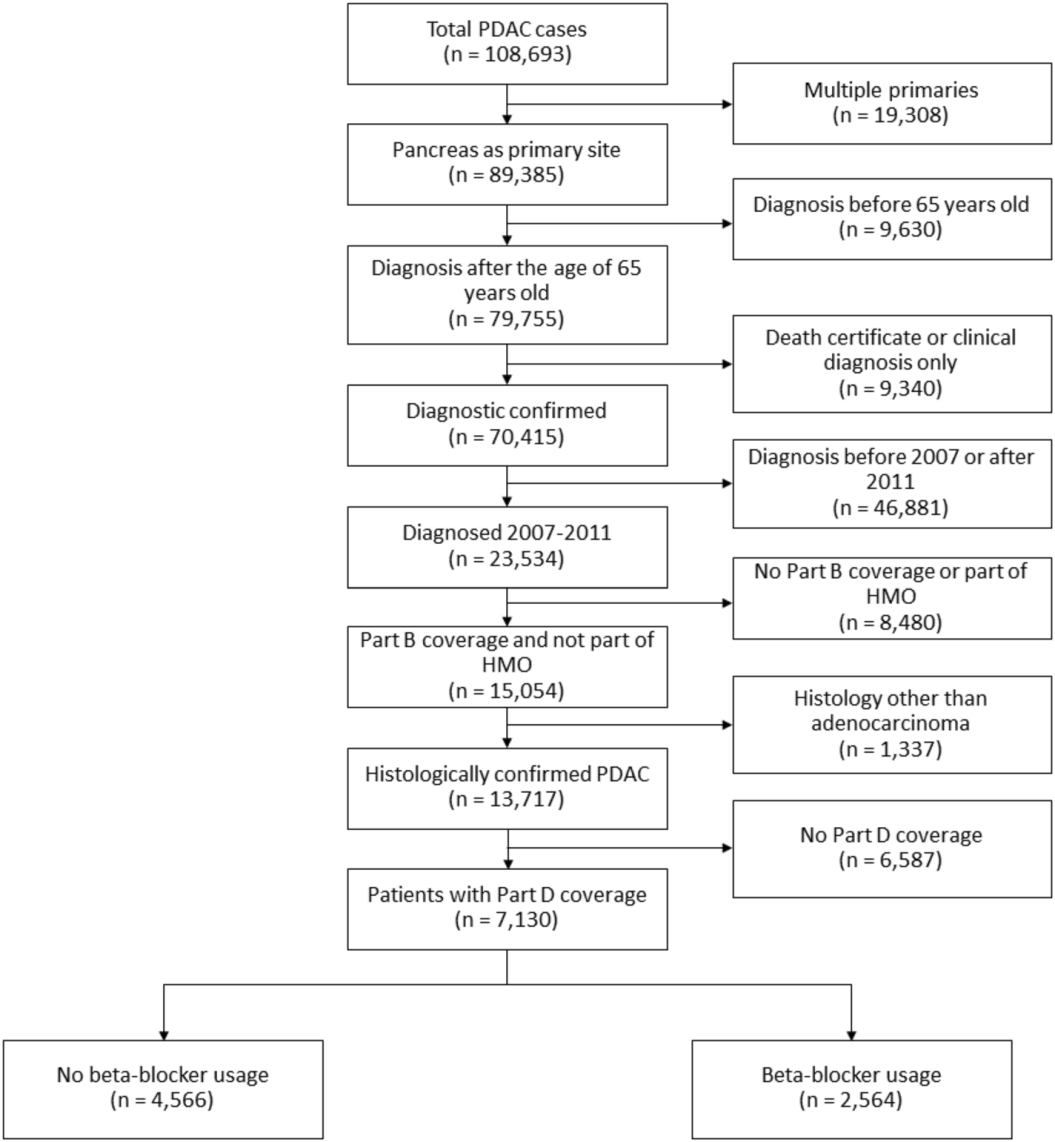
Cohort selection. *PDAC* pancreatic ductal adenocarcinoma, *HMO* health-care maintenance organization.

Patients aged 65 years and older with histologically confirmed PDAC diagnosed between 2007 and 2011 were selected (Fig. 1). The last date of diagnosis in our study was in December 2011 because that is when the verified dataset from SEER ended at the time this study was initiated. Only patients with one primary cancer were included to avoid the confounding effect of metachronous and synchronous lesions. PDAC was selected using the following International Classification of Diseases for Oncology, Second Edition (ICD-O-2) histology codes: 8000, 8010, 8140, 8500, 8550, and 8560. Patients without Medicare Part B were excluded to account for missing outpatient medical claims. Individuals in health-care maintenance organization (HMO) were also excluded to ensure all claims were captured. Next, we further limited patients by Medicare Part D coverage to account for prescription medication pharmacy claims (Fig. 1). For our primary analysis, we limited coverage to patients who had Medicare Part D in the 6 months prior to cancer diagnosis. As a subgroup analysis, we then further limited patients to Medicare Part D coverage in the 6 months before and 6 months after diagnosis so as to capture medication claims in the 12-month period surrounding cancer diagnosis.

All methods were carried out in accordance with relevant guidelines and regulations and all experimental protocols were approved by the Icahn School of Medicine at Mount Sinai Institutional Review Board (IRB). A waiver of informed consent was approved by the Icahn School of Medicine Mount Sinai IRB as this study was a retrospective chart review of a SEER-Medicare dataset and contains no personal identifiers.

### Covariates

Sociodemographic characteristics and clinical data were obtained from both the SEER registry and the Medicare claims data. Sociodemographic characteristics included age, sex, marital status, race and income. Income was derived by linking patients’ zip code to census data. Income was then dichotomized into four quartiles. The comborbid conditions needed for the Charlson comorbidity index were also collected^25^. Tumor and treatment characteristics included American Joint Committee on Cancer (AJCC) staging, cancer-directed surgery, radiation therapy and chemotherapy. Medication usage was identified using Medicare Part D claims. Beta-blocker usage within 6 months before and in the 12 month period surrounding the diagnosis of PDAC was identified. Beta-blockers considered were acebutolol, atenolol, bisoprolol, carvedilol, labetalol, metoprolol, nadolol, nebivolol, pindolol, propranolol, and sotalol. Beta-1 selective beta-blockers included acebutolol, atenolol, bisoprolol, metoprolol and nebivolol. All other beta-blockers were considered to be non-selective. Patients using a combination of both non-selective and selective beta-blockers were considered as using non-selective beta-blockers. Pre-existing conditions with indications for the use of beta-blockers were also identified. These included hypertension, arrhythmia, acute myocardial infarction, tachycardia, heart failure, angina pectoris, heart valve disease, coronary artery disease, cardiomyopathy and cirrhosis. Patients were considered to have these pre-existing conditions if there were at least two claims among inpatient and outpatient claims more than 6 months, but less than 2 years before PDAC diagnosis^26^.

### Outcomes

The primary outcome of interest was overall PDAC survival in patients who used beta blockers compared to patients who did not use beta blockers in the 6 months before cancer diagnosis. Survival time was defined as the time from the diagnosis of PDAC to death. Since pancreatic cancer has a high mortality rate, mortality was expected to reflect pancreatic cancer-related survival, as has been reported in other studies using SEER^17^. As a secondary analysis, we performed a subgroup analysis of patients with PDAC who had continuous Part D Medicare coverage in the 6 months before and after diagnosis to determine the overall survival effect of beta blocker use surrounding PDAC diagnosis.

### Statistical analysis

Chi-squared test and student t-test were used to compare the demographic characteristics between patients who used beta-blockers and those not using beta-blockers. The Kaplan–Meier method was used for overall survival analysis between patients using beta-blockers and patients not using beta-blockers.

We controlled for comorbid conditions and cancer-directed treatment modalities using a propensity score analysis. Propensity scores were used to adjust for potential confounding factors that may predispose usage of beta-blockers. Calculation of propensity scores was done using a logistic regression based on sociodemographic characteristics (sex, age, marital status, race, income) and Charlson comorbidity score. Cox proportional-hazards modeling was done with regression adjustment for confounders including propensity score, stage, cancer-directed surgery, radiation therapy and chemotherapy. All analyses were performed using SAS 9.4 (SAS Institute, Cary, NC).

### Ethical approval

All authors have approved the submitted version and have agreed both to be accountable for the authors’ own contributions and ensure that questions related to the accuracy or integrity to any part of the work, even ones in which the author was not personally involved, are appropriately investigated, resolved, and the resolution documented in the literature.

## Results

From 2007 to 2011, 13,731 patients were diagnosed with PDAC. Of these, 7130 patients had Medicare Part D coverage in the 6-month period before diagnosis (Table 1). 2564 (36%) patients were using a beta-blocker within 6 months before diagnosis. A majority (64%) of patients did not use a beta-blocker. Patients using a beta-blocker were more likely to be older, female, and have higher Charlson comorbidity scores (p < 0.01 for all). After propensity weighting for the likelihood of a patient receiving a beta-blocker, there were no significant differences in patient demographics.

**Table 1:** Patient demographics.

	No Beta-Blockers	Beta-Blockers	p-value	Adjusted p-value
N	4566 (64%)	2564 (36%)		
Mean age in years (standard deviation)	77.1 (7.9)	77.9 (7.5)	< 0.01	0.98
**Sex**				
Male	1838 (40.3%)	901 (35.1%)	< 0.01	0.96
Female	2728 (59.8%)	1663 (64.9%)		
**Marital status at diagnosis**				
Not married	2371 (53.8%)	1380 (56%)	0.08	0.99
Married	2034 (46.2%)	1084 (44%)		
**Race**				
Caucasian	3743 (82.1%)	2094 (82%)	0.58	0.93
African-American	449 (9.9%)	267 (10.5%)		
Other	365 (8%)	192 (7.5%)		
**Charlson comorbidity score**				
0	2027 (46.2%)	798 (31.3%)	< 0.01	0.47
1	1313 (29.9%)	776 (30.4%)		
2	533 (12.2%)	446 (17.5%)		
3 +	514 (11.7%)	534 (20.9%)		
**Income quartile**				
1	634 (13.9%)	356 (13.9%)	1	0.98
2	1297 (28.4%)	727 (28.4%)		
3	1552 (34%)	875 (34.1%)		
4	1083 (23.7%)	606 (23.6%)		

Adjusted p-value reflects the p-value after adjusting for propensity score for beta-blockers 6 months after diagnosis. Percentages or standard deviations are expressed in parentheses.

With regards to tumor characteristics, a majority of patients for both groups had an AJCC stage of IV (Table 2). Additionally, most patients did not receive any treatment, including surgery, chemotherapy and radiation. In terms of treatment, patients not using beta-blockers were more likely to receive cancer-directed surgery (15 vs 12%, p < 0.01). Radiation therapy and chemotherapy did not significantly differ between the two groups (p = 0.11).

**Table 2:** Tumor and Stage Characteristics for those using beta blockers prior to diagnosis.

Tumor/treatment characteristics	No beta-blockers	Beta-blockers	p-value
**American Joint Committee on cancer stage**			
I	307 (8%)	199 (9%)	0.38
II	1028 (26%)	594 (27%)	
III	336 (9%)	193 (9%)	
IV	2238 (57%)	1230 (56%)	
**Cancer-directed surgery**			
No	3844 (85%)	2235 (88%)	< 0.01
Yes	669 (15%)	295 (12%)	
**Radiation**			
No	3906 (87%)	2203 (87%)	0.47
Yes	595 (13%)	318 (13%)	
**Chemotherapy**			
No	2663 (58%)	1541 (60%)	0.14
Yes	1903 (42%)	1023 (40%)	
Survival time (months)	6.0 (8.5)	5.1 (7.5)	< 0.01

Percentages or standard deviations are expressed in parentheses.

**Figure 2:**
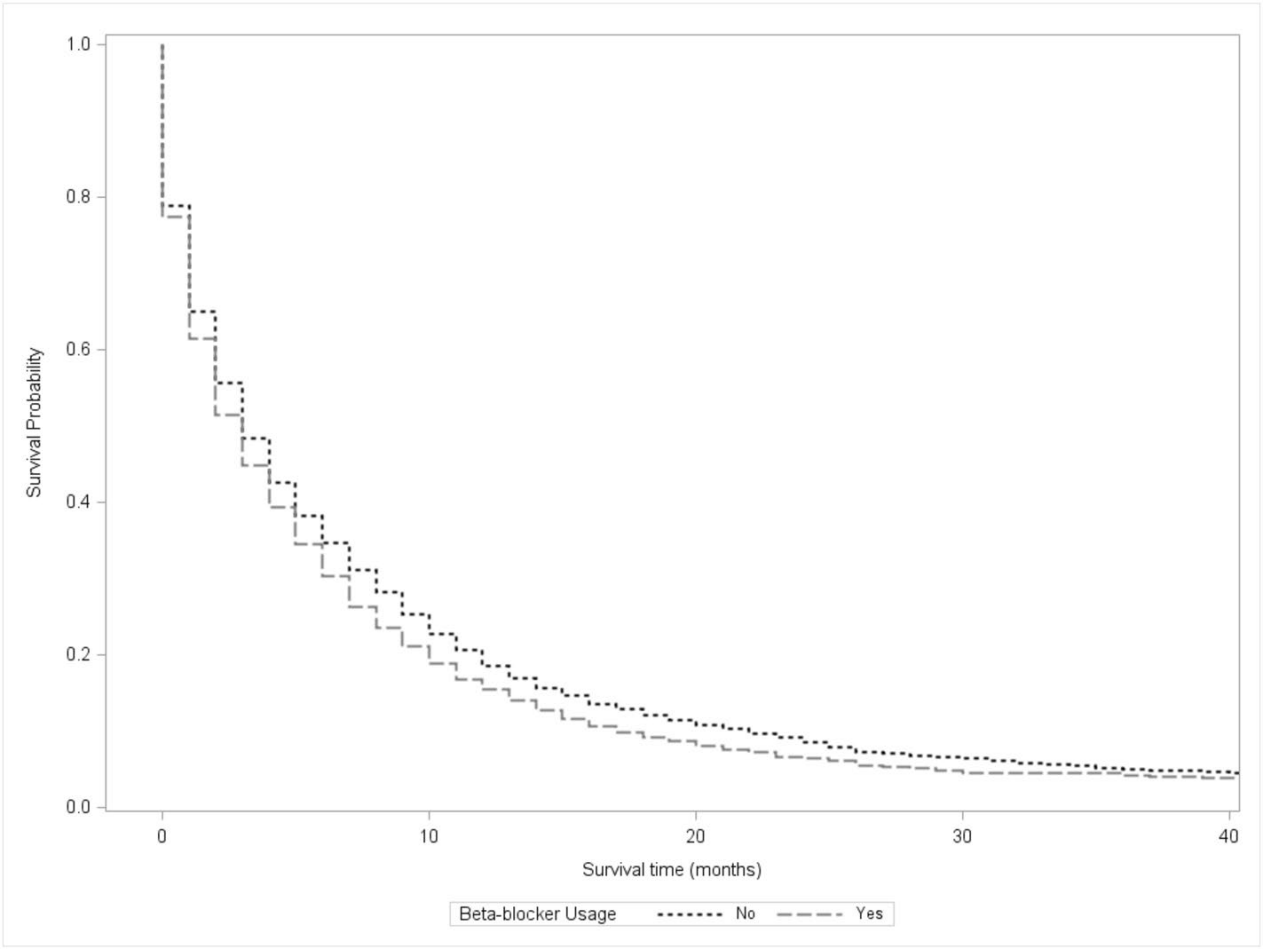
Kaplan–Meier Curve comparing survival of patient using beta-blockers 6 months before diagnosis to patients not using beta-blockers, p < 0.001.

On Kaplan–Meier analysis, patients not using beta-blockers had a longer overall survival (6 vs. 5.1 months, p < 0.01) (Fig. 2). After stratification by disease stage, this difference persisted for AJCC stages II-IV (p < 0.05), but did not persist for stage I (p = 0.14) (Supplemental Table S1). For patients not receiving any cancer-directed therapies (surgery, chemotherapy or radiation therapy), patients not using beta-blockers still demonstrated improved survival compared to patients who did not (p < 0.01). For patients receiving chemotherapy, patients not using beta-blockers demonstrated improved survival. However, for patients receiving cancer-directed surgery (p = 0.23) and radiation (p = 0.16), patients not using beta-blockers did not demonstrate improved survival.

After adjustment for confounding factors, including AJCC stage and cancer-directed treatment using the Cox-Proportional Hazard model, there were no differences in survival (Hazard Ratio (HR) 1.04, 95% Confidence Interval (CI) 0.98–1.1, p = 0.2) between patients who received beta-blockers and those who did not (Table 3). Diagnosis of advanced-stage cancer was associated with worse survival (p < 0.01 for stages II/III/IV). Cancer-directed surgery (HR 0.43, CI 0.38–0.48, p < 0.01), radiation therapy (HR 0.73, CI 0.66–0.80, p < 0.01) and chemotherapy (HR 0.39, CI 0.37–0.41, p < 0.01) were protective factors associated with better survival.

**Table 3:** Cox-Proportional Hazards model for beta-blockers use before diagnosis, adjusted for tumor stage, cancer-directed surgery, radiation therapy, chemotherapy and propensity score.

	Hazard Ratio (95% CI)	p-value
Beta-blocker	1.04 (0.98–1.1)	0.2
**Stage**		
I	Ref	
II	1.45 (1.28–1.64)	< 0.01
III	1.56 (1.35–1.8)	< 0.01
IV	2.48 (2.21–2.79)	< 0.01
Surgery	0.43 (0.38–0.48)	< 0.01
Radiation	0.73 (0.66–0.80)	< 0.01
Chemotherapy	0.39 (0.37–0.41)	< 0.01

Propensity score included sex, age, marital status, race, income and Charlson comorbidity score.

We then performed stratified analyses by receipt of cancer directed therapies (both chemotherapy and surgical resection) and no treatment. In these analyses, there was no survival advantage in patients who used beta-blockers in the 6 months preceding diagnosis. A further stratified analysis was performed limited to patients with early stage disease (AJCC stage I or II) who underwent chemotherapy and surgery. On Kaplan–Meier analysis, patients in this subgroup who did not use beta blockers survived 13.3 months (SD 12.4) compared to the 11.19 months (SD 11.7) of patients who used beta blockers (0.03), though this was no longer significant on multivariate analysis.

**Figure 3:**
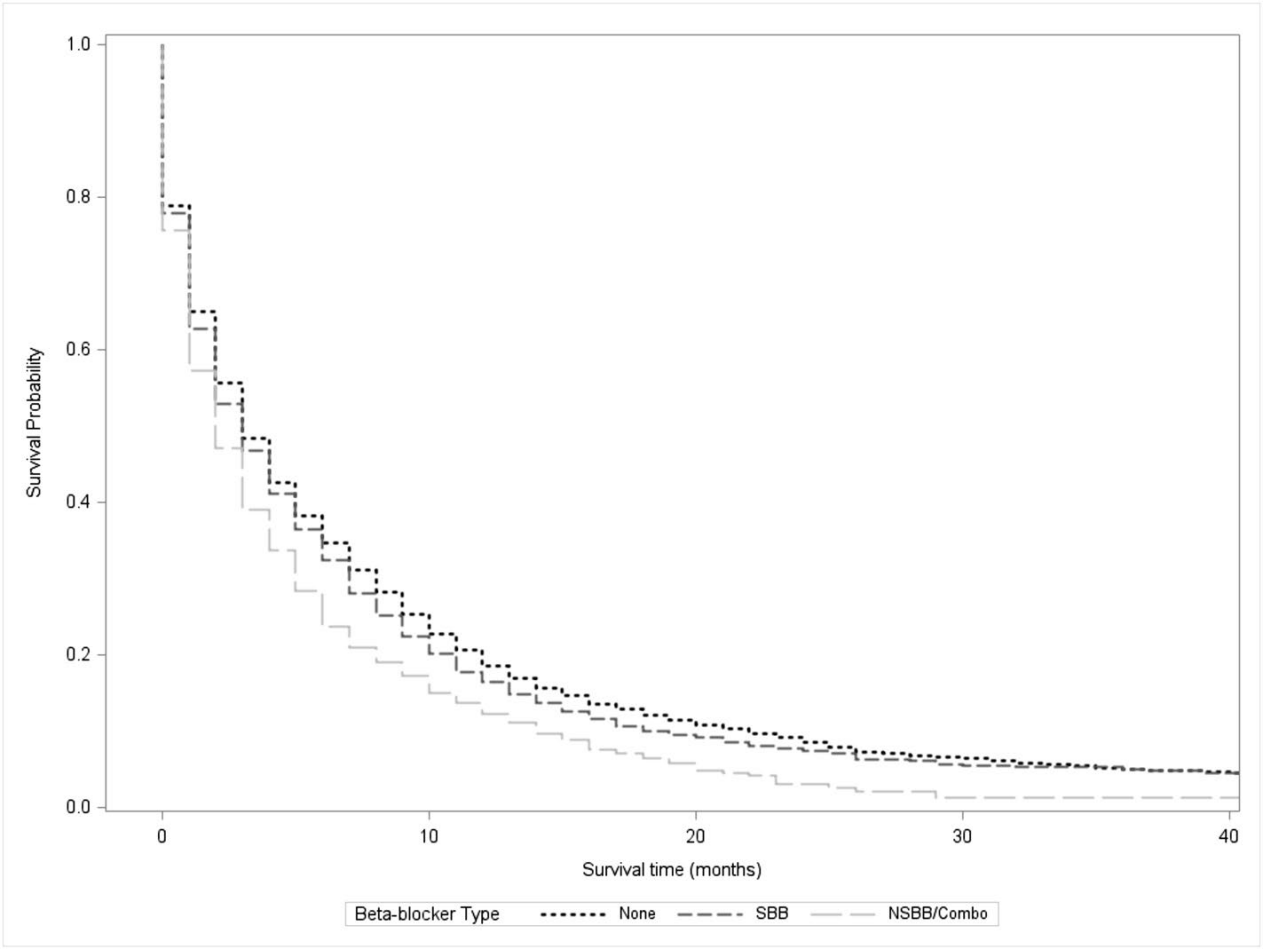
Kaplan–Meier Curve comparing the survival of patients using selective beta-blockers (SBB) and non-selective beta-blockers/combination (NSBB/Combo) to patients not using any beta-blockers (None), p < 0.001.

To better evaluate the effect of specific beta-blockers before diagnosis, beta-blockers were stratified into selective beta-blockers and non-selective beta-blockers/combination. Overall, 1956 (76%) patients used only selective beta-blockers before diagnosis, while 608 (24%) patients used non-selective beta-blockers or combinations of both selective and non-selective beta-blockers before diagnosis (Supplemental Table S2). After stratification into selective beta-blockers and non-selective beta-blockers/combination, univariable Kaplan–Meier analysis again demonstrated that patients using these medications before diagnosis had a significantly shorter overall survival time compared to those who did not receive any beta-blockers before diagnosis (5.4 months for selective beta blockers vs 4.1 months for nonselective beta-blockers vs 6 months for no beta blocker use, Fig. 3). With regards to selective beta-blockers, the majority of these patients used metoprolol (51%) or atenolol (24%). Patients using metoprolol before diagnosis (5.2 vs. 5.8 months, p = 0.01) had a shorter survival time compared to patients not using metoprolol. Patients receiving atenolol before diagnosis (5.7 vs. 5.7 months, p = 0.86) demonstrated no survival difference compared to those who did not use atenolol. For non-selective beta-blockers, most of these patients used carvedilol before diagnosis (16%). Patients using carvedilol before diagnosis also demonstrated shorter survival than patients who did not use carvedilol (4.3 vs. 5.7 months, p < 0.01).

These survival differences did not persist on multivariable analysis (HR for non-selective beta blockers: 1.07, 95% CI 0.97–1.19, p = 0.16; HR for selective beta-blockers: 1.02, 95% CI 0.96–1.09, p = 0.51) (Table 4). After stratification by indications for beta-blocker usage, patients using beta-blockers before diagnosis did not demonstrate significantly different survival times compared to those who did not use beta-blockers (p > 0.05 for all).

**Table 4:** Cox-Proportional Hazards model for beta-blockers, non-selective beta-blockers/combination (included concurrent usage of selective and non-selective beta-blockers) and selective beta-blockers prior to diagnosis stratified by condition, adjusted for tumor stage, cancer-directed surgery, radiation therapy, chemotherapy and propensity score.

	Beta-blockers	p-value	Non-selective beta-blockers/combination	p-value	Selective beta-blockers	p-value
	Hazard ratio (95% CI)		Hazard ratio (95% CI)		Hazard ratio (95% CI)	
Overall	1.04 (0.98–1.11)	0.18	1.07 (0.97–1.19)	0.16	1.02 (0.96–1.09)	0.51
Hypertension	1.03 (0.96–1.11)	0.46	1.05 (0.94–1.18)	0.41	1.01 (0.94–1.09)	0.78
Arrhythmia	1 (0.86–1.15)	0.95	1.01 (0.84–1.22)	0.9	0.99 (0.85–1.14)	0.86
Acute myocardial infarction	1.74 (0.77–3.94)	0.18	1.3 (0.53–3.21)	0.57	1.85 (0.82–4.16)	0.14
Tachycardia	1.01 (0.8–1.27)	0.94	0.97 (0.7–1.36)	0.87	1.06 (0.84–1.34)	0.64
Heart failure	1.14 (0.95–1.38)	0.17	1.11 (0.89–1.37)	0.36	1.08 (0.89–1.3)	0.46
Angina pectoris	0.79 (0.54–1.17)	0.24	1.16 (0.73–1.85)	0.53	0.72 (0.49–1.07)	0.11
Heart valve disease	1.02 (0.64–1.62)	0.94	0.59 (0.32–1.09)	0.09	1.34 (0.85–2.12)	0.21
Coronary artery disease	0.98 (0.86–1.12)	0.78	0.93 (0.78–1.1)	0.39	1.01 (0.88–1.15)	0.92
Cardiomyopathy	0.91 (0.61–1.37)	0.66	0.98 (0.66–1.46)	0.91	1.03 (0.69–1.54)	0.89
Cirrhosis	0.99 (0.52–1.89)	0.98	1.17 (0.5–2.78)	0.71	1.06 (0.54–2.11)	0.86

Propensity score included sex, age, marital status, race, income and Charlson comorbidity score. Reference for each group includes all other patients not using the specified medication.

As an additional subgroup analysis, we limited our set to include patients who had Part D Medicare in the 6-month period before and after diagnosis in order to evaluate survival in patients who used beta-blockers surrounding their PDAC diagnosis. We excluded 45 patients, which limited our set to 7085 patients. Of these patients, 1750 (24.7%) used beta-blockers both before and after cancer diagnosis. On Kaplan–Meier analysis, patients using beta-blocker during this prolonged time period had a longer overall survival: 6.4 (SD 7.9) vs. 5.4 months (8.3), p < 0.01. After adjustment for confounding factors, including AJCC stage and cancer-directed treatment using the Cox-Proportional Hazard model, beta-blocker use still showed improved survival (Hazard Ratio (HR) 0.86, 95% Confidence Interval (CI) 0.80–0.92, p < 0.01).

## Discussion

In this study utilizing a large claims database, beta-blocker usage in the 6 months before diagnosis was not associated with a survival advantage in patients diagnosed with PDAC. However, our study did find a survival advantage in patients who used beta-blockers within the 12-month period surrounding diagnosis. Our study is the first US-based epidemiological study to specifically demonstrate the lack survival benefit in patients with PDAC who used beta-blockers in the 6 months before diagnosis and to control for cancer directed therapies.

It has been theorized that beta-blockers may inhibit cancer advancement by decreasing catecholamine surges^8^, and pre-clinical trials have shown that beta-blockers can prevent cancer progression^7,10^. However, there are few clinical studies assessing survival in pancreatic cancer, and these studies have conflicting results and evaluate beta-blocker use at different periods throughout PDAC diagnosis^16,17,22,23^. A UK-based study by Shah et al. using the Doctors’ Independent Network database of multiple cancers demonstrated decreased survival for patients with PDAC who used beta-blockers in the 1-year period before diagnosis^22^. In our study we also initially found decreased survival in patients who used beta-blockers prior to diagnosis, though after we adjusted for cancer directed therapies no survival difference between the two groups was seen. Since Shah et al.’s study did not take into account cancer stage or cancer therapies^22^, it is likely that the decreased survival seen in patients who used beta-blockers was confounded by lack of cancer treatment, possibly due to the significant cardiovascular disease in patients who use beta-blockers. Additionally, Shah et al.’s control group comprised of patients with PDAC who used other anti-hypertensive agents^2^, and it is therefore possible that other agents among the many classes used to treat hypertension may exert its own effect on PDAC survival. Furthermore, another UK-based study, using the Clinical Practice Research Datalink database and the same analysis from Shah et al., refuted these results when databases where combined and found no association between survival and beta-blocker usage^23^. Lastly, both of these studies were limited by small sample sizes of fewer than 500 patients with PDAC^22,23^.

A third study by Udumyan et al., using the Swedish Cancer Registry of 2394 patients with PDAC demonstrated that beta-blockers conferred a survival benefit but they too did not account for treatment modalities, such as cancer-directed surgery, chemotherapy and radiation therapy^16^. Another possible reason for the difference in survival seen in our study and Udumyan et al.’s, is that our study contained an older population (our mean age was 77 years compared to Udumyan et al.’s of 70 years), and later stage disease (55% of our study had stage IV disease compared to 30% in Udumyan’s study), making our sample a sicker overall cohort who may have worse overall comorbidities impacting survival^16^. Udumayan et al. found the greatest survival benefit in patients with non-metastatic disease^16^. However, when limiting our cohort to individuals with early stage disease (stage I or II) who received chemotherapy and surgical resection, beta-blocker usage was not associated with improved survival on multivariate analysis. It is possible then that any survival impact seen in patients who use beta-blockers may no longer apply when they undergo cancer directed therapies.

Since pre-clinical studies suggest that non-selective beta-blockers may have a greater effect on inhibiting cancer progression compared to selective beta-blockers^7^, we evaluated if type of beta-blocker use before diagnosis impacted survival. We found no survival benefit in patients who used either selective or non-selective beta-blockers prior to diagnosis. While non-selective beta-blockers may have a greater benefit than selective beta-blockers in ovarian cancer^14^, other studies in pancreatic cancer found no clear difference between type of beta-blocker^16^. We also stratified beta-blocker use before diagnosis by pre-existing indication for beta-blocker use, but did not find a survival advantage for any patient subgroup. This was true even when simultaneously evaluating the effect of selective beta-blockers and non-selective beta-blockers or combination.

In a subgroup analysis evaluating patients with beta-blocker use in the 12-month period surrounding diagnosis, we did find a significant improvement in survival in patients who used beta-blockers. Our finding corroborates a 2017 study done by Beg et al. that demonstrated a beneficial effect of beta-blockers on overall survival in patients using beta-blockers within 12 months of PDAC diagnosis using the SEER registry between 2007 and 2009^17^. Our study further supports the possible impact of continuous beta-blocker use as we saw a benefit even after adjusting for cancer stage and cancer directed therapies which was not done and therefore a limitation of Beg et al.’s earlier study^17^. It is possible that beta-blockers may confer a survival advantage by impacting tumor signaling pathways over a prolonged time period surrounding diagnosis. On the other hand, other factors, such as improved access to the healthcare system or continued use of cardiac medications among patients who used beta-blockers over a longer period of time may have played a role. Furthermore, while the HRs were significant, mean survival time was quite poor overall, suggesting that the difference in survival may have signified a difference as little as a few days.

There are several limitations with this study. This study is a retrospective analysis of a large database, which has inherent limitations associated with such a study design. Because the database is dependent on physician reporting and proper coding, the accuracy of the data is subject to the quality of the reporting. Additionally, beta-blocker use was determined by Medicare claims data from Part D, which includes medications claims data and does not necessarily correspond with medication compliance. However, prior studies have shown that claims data correlates with medication use in an elderly population^27^. We were also unable to control for the dosage of medications, as these data are not available. Important prognostic factors such as smoking status and CA 19-9 levels are also not available and could not be included in our analyses^28,29^. Additionally, we did not assess for the presence of other medications’ impact on survival associated with PDAC. Although there are conflicting data, it has been suggested that several other medications, including metformin, insulin and statins, may impact the survival associated with PDAC and consequently, the results of this study^17,26,30,31^. PDAC is also heterogeneous with some malignancies demonstrating resistance to treatment such as chemotherapy, which could not be controlled for in this study^32^. Since the SEER-Medicare database is reliant solely on Medicare data, it is possible that access to care plays an important role in survival, as patients with private insurance may have had earlier diagnosis which may have improved overall survival. Finally, the differences in the management of PDAC can vary widely across the country and may also affect the survival of patients^33^.

In conclusion, our study found no difference in survival in patients who used beta-blockers prior to PDAC diagnosis, but improved survival in patients who used beta-blocker within 12 months of PDAC diagnosis. The lack of survival advantage in patients who used beta-blockers prior to diagnosis persisted through stratification by beta-blocker indications and selectivity of the beta-blocker. To our knowledge, this is the first US study to evaluate survival in patients who use beta-blockers prior to PDAC diagnosis, and the largest dedicated US study to evaluate survival in patients with PDAC who use beta-blockers that also controls for cancer-directed therapies and comorbid conditions. Further prospective studies are needed to determine the role of beta-blockers in PDAC.

## Supplementary Information

Supplementary Information.

## Data Availability

The datasets used and/or analyzed during the current study are available from the National Cancer Institute.
