# Supplementary material for: Beta-blockers have no impact on survival in pancreatic ductal adenocarcinoma prior to cancer diagnosis
Source: Sci Rep. 2021 Jan 13;11:1038. doi: 10.1038/s41598-020-79999-0 (PMC7807087; doi:10.1038/s41598-020-79999-0)
Supplement: Supplementary file 1 — Supplementary Information. [file 41598_2020_79999_MOESM1_ESM.docx]

Appendix:

Supplemental Table 1. Mean overall survival by cancer stage and therapy in patients who used beta-blockers in pre diagnosis compared to patients who did not use beta-blockers during this time period. Standard deviations are expressed in parentheses.

| Survival Time (months) |  | No Beta-Blocker Use | Beta-Blockers | p-value |
| --- | --- | --- | --- | --- |
| Overall |  | 6 (8.5) | 5.1 (7.5) | <0.01 |
| American Joint Committee on Cancer stage | I | 9.2 (10.9) | 7.8 (10.2) | 0.2 |
|  | II | 10.4 (10.9) | 8.7 (9.7) | <0.01 |
|  | III | 7.5 (7.6) | 6.1 (6.5) | 0.01 |
|  | IV | 3.4 (5.2) | 3.1 (4.9) | 0.01 |
| Cancer-directed surgery | No | 4.5 (6.5) | 4.1 (5.9) | <0.01 |
|  | Yes | 14.5 (12.7) | 13.1 (12.2) | 0.23 |
| Radiation | No | 5.1 (7.8) | 4.3 (6.7) | <0.01 |
|  | Yes | 11.8 (10.4) | 10.9 (10.1) | 0.16 |
| Chemotherapy | No | 3.5 (6.9) | 2.8 (5.5) | <0.01 |
|  | Yes | 9.5 (9.2) | 8.5 (8.7) | 0.01 |

Supplemental Table 2. Mean Survival (months) comparing categories and types of beta-blocker use prior to diagnosis, unadjusted analysis.

|  | Number of patients | No medication | Medication | Log-rank p-value |
| --- | --- | --- | --- | --- |
| Beta blockers | 2564 (100%) | 6 (8.5) | 5.1 (7.5) | <0.01 |
| Selective beta-blockers | 1956 (76%) | 5.8 (8.2) | 5.4 (7.9) | 0.05 |
| Non-selective beta blockers/Combination | 608 (24%) | 5.8 (8.3) | 4.1 (5.7) | <0.01 |
| Acebutolol | ** (**%) | 5.7 (8.2) | 6.5 (5.9) | 0.76 |
| Atenolol | 620 (24%) | 5.7 (8.2) | 5.7 (8.1) | 0.86 |
| Bisoprolol | 59 (2%) | 5.7 (8.2) | 5.8 (7.3) | 0.42 |
| Carvedilol | 409 (16%) | 5.7 (8.3) | 4.3 (6.2) | <0.01 |
| Labetalol | 36 (1%) | 5.7 (8.2) | 3 (3.5) | <0.01 |
| Metoprolol | 1318 (51%) | 5.8 (8.2) | 5.2 (8) | 0.01 |
| Nadolol | 24 (1%) | 5.7 (8.2) | 4 (4.8) | 0.04 |
| Nebivolol | 36 (1%) | 5.7 (8.2) | 3.9 (4.1) | 0.04 |
| Pindolol | ** (**%) | 5.7 (8.2) | 4.3 (7.8) | 0.41 |
| Propranolol | 80 (3%) | 5.7 (8.2) | 3.9 (4.5) | <0.01 |
| Sotalol | 67 (3%) | 5.7 (8.2) | 4.1 (5) | 0.02 |

**data not reported for values <11, per SEER guideline
